# Improvement of Mitral Regurgitation after Transapical Transcatheter Aortic Valve Bioprosthesis Implantation

**DOI:** 10.1186/1749-8090-10-S1-A148

**Published:** 2015-12-16

**Authors:** Heraldo G Lobo Filho, José Glauco Lobo Filho, Nathalia RP Sousa, Matheus D Pimentel, Camylla S Souza

**Affiliations:** 1 Department of Surgery, Faculdade de Medicina da Universidade Federal do Ceará, Fortaleza, Brazil

## Background/Introduction

Implantation of transcatheter aortic bioprosthesis has become a procedure with increasingly relevance in medical practice for the treatment of aortic stenosis, especially in patients with high or prohibitive surgical risk for traditional surgery. The presence of mitral regurgitation (MR) may be associated with increased morbidity and mortality, however, some studies have shown reduction in the degree of regurgitation of this valve after treatment of the aortic valve.

## Aims/Objectives

To evaluate MR evolution in three patients with moderate to severe MR, and who underwent transcatheter implantation of aortic bioprosthesis trough transapical approach due to severe aortic stenosis.

## Method

Data were collected from medical records and databases. Of the 22 patients undergoing transcatheter implantation of aortic bioprosthesis trough transapical approach, due to severe aortic stenosis, in our service from May 2012 to May 2015, four were presenting with MR of moderate to severe before surgery. In one of these cases, we had no access to the postoperative echocardiogram. Regarding the group of three patients, two were females, mean age was 80.33 ± 7.63 years, Euroscore II average of 11.25 ± 3.1, STS-average score of 15.06 ± 9 26.

## Results

None of the three patients had complications in the procedure or during the immediate postoperative period, finding themselves alive, and in ambulatorial follow-up, with an average follow-up time of 19.33 ± 14.84 months. Echocardiography before hospital discharge, and outpatient follow-up showed aortic valve prostheses normal functioning, medium transprosthetic gradient less than 10 mmHg, and mild mitral insufficiency. Only one patient (33%) showed discreet paravalvular reflux.

## Discussion/Conclusion

In this study, patients undergoing transcatheter implantation of aortic bioprosthesis trough transapical approach, due to severe aortic stenosis, which had preoperative moderate or severe MR, demonstrated good outcome in the postoperative period and in ambulatorial follow-up, as well as significant reduction in mitral valve regurgitation.

